# The Microbial Diversity on the Surface of Smear-Ripened Cheeses and Its Impact on Cheese Quality and Safety

**DOI:** 10.3390/foods13020214

**Published:** 2024-01-10

**Authors:** Jasmine S. Ritschard, Markus Schuppler

**Affiliations:** Laboratory of Food Microbiology, Institute of Food, Nutrition and Health, ETH Zurich, Schmelzbergstrasse 7, 8092 Zurich, Switzerland; jasmine.ritschard@outlook.com

**Keywords:** smear-ripened cheese, surface-ripened cheese, washed-rind cheese, surface smear, red-smear, surface microbiota, microbial diversity, cheese microbiome

## Abstract

Smear-ripened cheeses are characterized by a viscous, red-orange surface smear on their rind. It is the complex surface microbiota on the cheese rind that is responsible for the characteristic appearance of this cheese type, but also for the wide range of flavors and textures of the many varieties of smear-ripened cheeses. The surface smear microbiota also represents an important line of defense against the colonization with undesirable microorganisms through various types of interaction, such as competitive exclusion or production of antimicrobial substances. Predominant members of the surface smear microbiota are salt-tolerant yeast and bacteria of the phyla Actinobacteria, Firmicutes, and Proteobacteria. In the past, classical culture-based approaches already shed light on the composition and succession of microorganisms and their individual contribution to the typicity of this cheese type. However, during the last decade, the introduction and application of novel molecular approaches with high-resolution power provided further in-depth analysis and, thus, a much more detailed view of the composition, structure, and diversity of the cheese smear microbiota. This led to abundant novel knowledge, such as the identification of so far unknown community members. Hence, this review is summarizing the current knowledge of the diversity of the surface smear microbiota and its contribution to the quality and safety of smear-ripened cheese. If the succession or composition of the surface-smear microbiota is disturbed, cheese smear defects might occur, which may promote food safety issues. Hence, the discussion of cheese smear defects in the context of an increased understanding of the intricate surface smear ecosystem in this review may not only help in troubleshooting and quality control but also paves the way for innovations that can lead to safer, more consistent, and higher-quality smear-ripened cheeses.

## 1. Introduction

The production of the fermented milk-based food product, cheese, not only allows the biopreservation of a nutritious food product in a transportable form for human nourishment, but also renders milk to an enhanced digestible food product [1,2,3]. Cheese in general poses a high nutritive value for human nourishment originating from its high proteins, fats, vitamins (vitamin A, riboflavin, vitamin B_12_, and to a lesser extent, folate), essential amino acids, minerals (calcium, phosphorous, magnesium, little iron, and sodium chloride) and low carbohydrate (trace amounts) content [4,5,6]. For the whole process of transformation of milk into cheese microorganisms play essential roles during all steps of production and maturation and provide an important contribution to the development of organoleptic properties through their metabolism and various enzymatic activities [7]. It is hardly surprising that today cheese is produced in a wide range of forms and flavors throughout the world and developed to a premium food product [4]. According to the Codex Alimentarius commission established by the FAO (Food and Agriculture Organization) and the WHO (World Health Organization), cheese is today defined in the international food standards as: “…*the ripened or unripened soft or semi-hard, hard or extra-hard product, which may be coated, and in which the whey protein/casein ratio does not exceed that of milk,* …” [8]. For cow’s milk this whey protein/casein ratio is typically 20/80, whereas in cheese up to 99% of the protein might be casein due to the effect of syneresis. During cheese production the amount of casein is increased, whereas an amount of whey proteins will be rejected while withdrawing the whey liquid, leading to a diminished whey protein/casein ratio.

### 1.1. Cheese Varieties

Hitherto, more than 1000 different cheese varieties are described and can be classified either based on their textural properties (very hard, hard, semi-hard, semi-soft, soft), on the method of coagulation (rennet, acid, acid/heat), on the milk type applied, on the cooking temperature, on the cheese composition, on the characteristic ripening agent, or on the ripening indices depicted according to the extent of chemical breakdown during cheese ripening [4,9,10]. Hitherto, no global classification system for cheese varieties exists, but in their review, McSweeney and colleagues grouped the principal categories of cheese accordingly [9,10].

Alpine cheeses are characterized by large cheese wheel sizes, hard rinds, and a smooth and elastic texture with the presence of eyes in the cheese matrix [9]. From the milk produced by grazing cows living on the limited land in the mountains, alpine herdsmen collaboratively produced cheese as non-perishable food for the harsh wintertime. In copper cauldrons, milk was cooked at high temperatures, the curd cut, and afterward pressed and ripened for several months, whereby all these procedures led to a facilitated whey expulsion and moisture loss. Thereby the slow delayed acid production remaining a high pH facilitated a high mineral content and led to a sweet cheese. This cheese production process resulted in hard, durable cheese, which is low in moisture. Therefore, this cheese variety has a long shelf-life and can be transported from the mountains to the valley [9].

In contrast, soft-ripened cheese varieties were intended for home consumption or sale in local villages and did not need to withstand prolonged storage periods or long transport routes. These cheese varieties are typically smaller in size and have a soft texture. Bloomy-rind soft cheeses such as Brie or Camembert originated in France and are characterized by the growth of mold such as *Penicillium camemberti* on their surface [9]. Extensively metabolized lactate on the surface of this cheese type creates a pH gradient and leads to the migration of lactate from the cheese core to the surface, which is followed by calcium phosphate precipitation that causes softening of the cheese [10]. 

Furthermore, there are smear-ripened cheeses, which are reviewed here in detail, such as Münster, Limburger, or Tête de Moine, that evolved within the monasteries in Europe. This type of cheese was originally produced for consumption within monasteries and succeeded as a meat substitute in some monastic orders, where meat consumption was banned [9]. Smear-ripened cheeses are characterized by the growth of a complex, mainly Gram-positive, bacterial microbiota on the cheese surface [10,11]. A recent review by Pepi and colleagues describes the production and microbiology of different cheese varieties in general, with a focus on critical production steps to ensure health, quality, and typicity of cheese and further dairy products [7]. Hence, this review will focus on smear-ripened cheese and the diversity of their surface-smear microbiota that contributes to flavor, appearance, safety, and typicity of this cheese variety.

### 1.2. Smear-Ripened Cheese

Smear-ripened cheeses are characterized by the development of a viscous, red-orange surface smear on their rind during ripening. This surface smear harbors the desired community of specific microorganisms, which are involved in the ripening process. They derive mainly from the facility-specific cheese-making environment such as the aging surfaces, the brine bath, or the air comprised in the ripening cellar and are collectively termed ‘house microbiota’ [12,13,14,15]. The multi-microbial species ecosystem harbored by the surface smear is mainly inhabited by Gram-positive bacteria and, to a lesser extent, by yeast and mold [11,16,17], but more recent studies also highlight the contribution of Gram-negative bacteria to the production of surface-ripened cheeses. The typical surface microbiota is usually dominated by salt-tolerant yeast (e.g., *Debaryomyces hansenii*, *Geotrichum candidum,* and *Candida* spp.) and ‘coryneform bacteria’ such as *Corynebacterium* spp., *Brevibacterium* spp., *Arthrobacter* spp., *Glutamicibacter* spp., and *Microbacterium* spp. together with *Staphylococcus* spp. and some Gram-negative bacteria, including *Halomonas* spp. and *Psychrobacter* spp. [18,19]. Therefore, this type of cheese is also known as ‘Bacterial surface-ripened cheese’ [11]. Further designations used synonymously for this cheese type comprise ‘Surface-ripened cheese’ and ‘Washed-rind cheese’ [20]. Matured cheeses develop a red-orange, viscous, and glistening smear on the surface and are therefore also termed ‘Red-smear cheese’ in the current linguistic usage [11,21]. As this type of cheese is usually produced as small cylindrical wheels, the resultant high surface area to volume ratio allows the surface microbiota to exert a direct impact on the flavor and texture characteristics of the mature cheese [10,11]. The moisture content of these typically semi-hard or soft cheeses is generally medium to high, which favors the diffusion of enzymes and biochemical compounds into the cheese interior, thus promoting the ripening process [22]. High levels of proteolysis and lipolysis, mainly at the cheese surface, result in a strong aroma, which is characteristic of this type of cheese [10,11]. Key compounds leading to an intense cheese flavor with garlic notes seem to be volatile aromatic sulfur compounds [11,16]. Highly concentrated at the cheese surface, sulfur compounds are produced mainly from methionine and cysteine during the cheese ripening process by the biochemical activity of the surface smear microbiota [11,16,23]. 

Surface-ripened cheeses have a long tradition. In earlier times they were produced without any scientific knowledge of the biochemical processes and the indigenous microbiota that contribute to the typical characteristics of this type of cheese [17,20,24]. Until quite recently, the making of cheese was considered to be an art rather than a science [4]. The traditional way of producing such cheese is called ‘old-young smearing’ because surface smear is transferred by washing matured (‘old’) cheeses with a saline solution and the subsequent use of this saline solution to wash freshly produced green (‘young’) cheeses [11,24]. Thereby, the typical surface smear microorganisms harbored by the smear of the mature cheeses are recycled and ensure the inoculation of the green cheese surface by the desired smear microorganisms required for surface ripening [11]. However, not only the desired typical surface smear microorganisms might be transferred. Undesired microorganisms like human pathogens, such as *Listeria monocytogenes*, or spoilage microorganisms, mainly mold, might also be transferred from mature cheeses to freshly produced green cheeses using this methodology [11,16,24,25,26]. In particular, the foodborne pathogen *Listeria monocytogenes* represents a noticeable threat to this type of cheese. Not only because ‘old-young smearing’ does increase the risk of contamination but also because *Listeria monocytogenes* may develop well on the surface of red-smear cheeses, which offer favorable conditions due to their physicochemical traits [27]. As softer cheese types like smear-ripened cheeses are more susceptible to contamination and growth of *Listeria monocytogenes*, and smearing machines were found to be an important source for *Listeria monocytogenes* contamination, food safety problems appear occasionally in this type of cheese [16,28,29,30,31]. Due to the hazard of spreading foodborne pathogens from ripened cheese to newly produced cheese, the traditional ‘old-young smearing’ technique is criticized by many food safety authorities and is, therefore, less often applied [11,16,32,33]. Nowadays, the equipment for washing cheeses, such as brushes, is cleaned and the saline solution is renewed before washing cheeses of differing ripening ages. Furthermore, surface starter cultures which are intended to support the development of a proper surface microflora are commercially available and will likely replace ‘old-young smearing’ in the future [16,24,34,35].

Today, bacterial surface-ripened red-smear cheese varieties are extensively produced in Europe, mainly in Austria, Belgium, Germany, and France, as well as in Switzerland (Table 1) [9,11,36]. Well known red-smear cheese varieties in Europe include Comté, Limburger, Münster, Pont-l’Évêque, Port de Salut, Reblochon, Romadur, Saint Paulin, Taleggio, Trappiste and Vacherin Mont-d’Or [9,11,16]. Popular and produced in Switzerland are further red-smear cheese varieties such as Appenzeller, Mutschli, Raclette cheese, Tête de Moine, or Tilsit. Many of the different red-smear cheese varieties produced in Europe are manufactured in distinct artisanal farmhouse environments and small-sized traditional village cheese dairies. In countries such as Germany, France, or Denmark, the production of red-smear cheese is more industrialized, and rather medium-sized cheese dairies are involved [32,37]. Differences in the applied raw material, the production manner, cheese curing techniques and the ripening process lead to a distinctive flavor and appearance for each of these cheese varieties [9,11]. 

## 2. The Manufacture of Smear-Ripened Cheese

Smear-ripened cheese can be produced from any kind of rennet curd [32]. To begin with the primary raw material, the microbial and chemical quality of the milk used for cheese production is crucial for the quality of the final cheese product [4]. Mostly bovine milk is applied for cheese production, and less frequent is ovine, caprine, or buffalo milk [10]. Genetic or physiological factors of the dairy animal as well as environmental factors like the feed or the climate can influence the milk composition. However, variations in milk composition are even country- or region-dependent [39].

**Figure 1:**
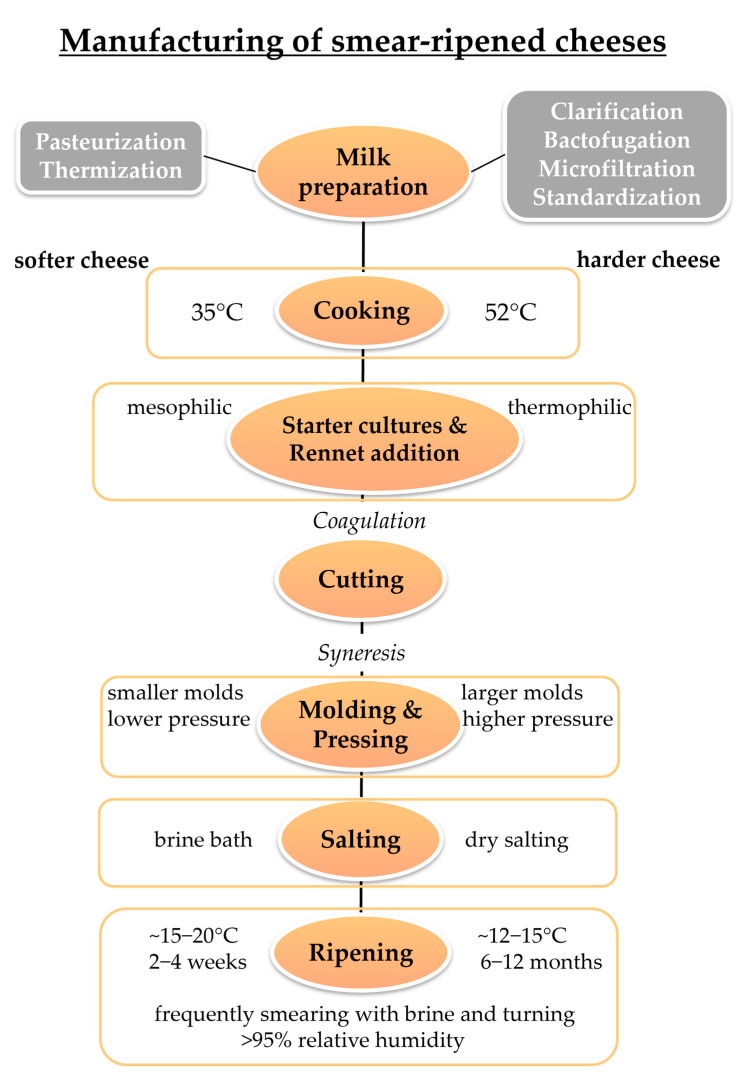
Typical processing steps for the production of surface-ripened red-smear cheese comprise heating of the milk, addition of starter cultures, molding and pressing of the curd with subsequent salting, as well as the final ripening process under environmental conditions that differ for the production of cheese with softer or harder final texture.

The typical processing steps for the production of surface-ripened red-smear cheese are shown in Figure 1. The first basic step during cheese manufacture is the preparation of the cheese milk. Milk can be clarified to eliminate foreign particles such as straw residues or leucocytes and epithelial cells from animals. High numbers of spores, in particular from *Clostridium* spp., present in the milk have to be reduced by microfiltration or bactofugation, a special centrifugation technique for milk [4,39]. To ensure important cheese compositional factors legally prescribed for the final cheese product, the composition of milk needs to be adjusted to the desired values of fat and protein prior to cheese curdling by standardization [4]. To reduce the amount of indigenous spoilage microorganisms present in the milk and to minimize the risk for transfer of vegetative pathogenic microorganisms to consumers, cheese milk is often pasteurized prior to the production of cheese. Pasteurization is a heat treatment procedure at 72 °C for 15 s or 63 °C for 30 min, as legally prescribed in the United States (US) and European Union (EU), used to reduce and standardize the microbial composition in milk. A proper pasteurization can be approved by a negative alkaline phosphatase test [9]. However, cheese produced from pasteurized milk often develops a less intense flavor and receives fewer scores for certain sensorial attributes. Furthermore, the ripening proceeds more slowly in comparison to raw milk cheese. Therefore, raw milk is still broadly used for the production of various traditional cheese varieties [4,40]. Thermization is also affecting the raw milk flora, but in a milder way than pasteurization, and is nowadays often applied as a preventive measure to control pathogens in raw milk [41]. Although thermization is a milder heat treatment at 60 °C or 67 °C for 30 s, it can still significantly reduce the total number of bacteria in raw milk. Therefore, it represents an important control measure for undesirable Gram-negative psychrotrophic bacteria, enterobacteria, and Gram-positive pathogens such as coagulase positive staphylococci and other pathogenic bacteria like *Listeria monocytogenes* [4,41].

In the second step, the prepared cheese milk is heated to a low temperature, usually below 35 °C [11]. Thereafter, mesophilic mixed-strain cultures of lactic acid bacteria (LAB) and rennet are used as starters for curdling of the milk, which means the conversion of liquid milk to cheese curd [4,11,42]. Such mesophilic mixed-strains cultures may contain two to six phage-unrelated defined strains of *Lactococcus lactis* subsp. *lactis* or *Lactococcus lactis* subsp. *cremoris*, which produce reproducible rates of lactic acid during the cheese milk fermentation [4]. The key step in the manufacture of good quality cheese is acid production at an appropriate rate and time, as it affects the coagulant activity and coagulum strength. However, the rate of proteolysis during ripening is also directly influencing the bacterial growth and enzymatic activity. Furthermore, the acidification controls the growth of pathogenic or gas-producing microorganisms and therefore contributes to the safety and quality of the final cheese [4]. The acidification by the starter cultures and the enzymatic activity of the rennet destabilizes the casein micelles in the milk, which next form a network termed coagulum or gel. The coagulation of the cheese milk occurs quickly when cheese milk comes to a still-stand [4,42]. Parameters such as the milk composition, technological factors affecting the cheese milk (prior heat treatment or refrigeration, homogenization or ultrafiltration), and coagulation process conditions (rennet enzyme concentration, temperature and pH during coagulation) affect the rennet coagulation time and the clotting time [42].

Post-coagulation, when the coagulum has attained sufficient firmness, the curd is cut into grains [4,42]. The smaller the coagulum is cut into grains, the more the syneresis is promoted, which is the exudation of whey when the curd contracts [10,43]. The shrinkage of the gel network proceeds slowly, and the drainage determines the final moisture content of a cheese. Mechanisms responsible for syneresis include the shrinkage and rearrangement of casein micelles and a decrease in solvation [43]. Further steps to separate the whey from curd involve molding and pressing, whereby in total a gel might lose up to 90% of its volume [43]. For the production of most smear-ripened cheese varieties, the cutting of the rennet coagulum may be delayed, and the curd reveals poor syneresis properties. The curd is then cut into large particles and molded as cheese wheels of rather small dimensions, which are only lightly pressed. Consequently, smear-ripened cheese curd retains whey very well, which is why this kind of cheese has a high moisture content and displays a soft or semi-hard texture [22]. Exceptionally, in the case of harder smear-ripened cheese varieties, such as Gruyère, Comté, or Beaufort, the procedure of cheese production is slightly different from the one described for soft and semi-hard smear-ripened cheese. Instead of mesophilic starter cultures, rather thermophilic starter cultures composed of *Streptococcus salivarius* subsp. *thermophilus*, *Lactobacillus helveticus*, *Lactobacillus delbrueckii* subsp. *lactis*, *Lactobacillus delbrueckii* subsp. *bulgaricus,* and/or *Lactobacillus delbrueckii* subsp. *casei* are applied for curdling of the milk [4,11]. Furthermore, the cheese milk is heated to higher temperatures than 35 °C and cheese curd is pressed in a large sized cheese mold at a high pressure after syneresis. Consequently, smear-ripened cheeses such as Gruyère have a low moisture content [11]. Finally, besides soft cheese varieties, smear-ripened cheese can comprise semi-soft (45–55% moisture content), semi-hard (45–50% moisture content), and hard (35–45% moisture content) smear-ripened cheese types [32].

Salting cheese in a brine bath is the last manufacturing step before the ripening process starts [4]. Besides the contribution of syneresis to reduce moisture, the main effects of salting cheese are the following: Control of microbial growth, microbial activity, and enzymatic activities in cheese or physical changes influencing cheese texture, protein solubility, and protein conformation. In addition to food preservation strategies, the salting of cheese contributes largely to the flavor and taste characteristics of a cheese [44]. Cheese wheels are immersed in a brine bath of 19–21% (*w*/*w*) sodium chloride concentration during 4–18 h at a temperature of 10–14 °C and are thereafter air-dried [11,39]. As the brine bath is contaminated by cheese particles, proteins, and microorganisms during this step, filtration, clarification, or pasteurization of the brine from time to time is essential to maintain a high brine quality [39]. Alternatively, harder surface-ripened cheese varieties may be rubbed with dry salt for several times during ripening [11].

Variances in the cheese curds after production relate mainly to variations in cheese milk composition and cheese manufacturing techniques. Cheese composition and texture, predetermined by e.g., moisture, sodium chloride content, pH, and the starter culture, affect the following ripening period. Nevertheless, the unique characteristics of the individual cheese varieties are principally established during the cheese ripening process [4].

## 3. The Ripening of Smear-Ripened Cheese

**Figure 2:**
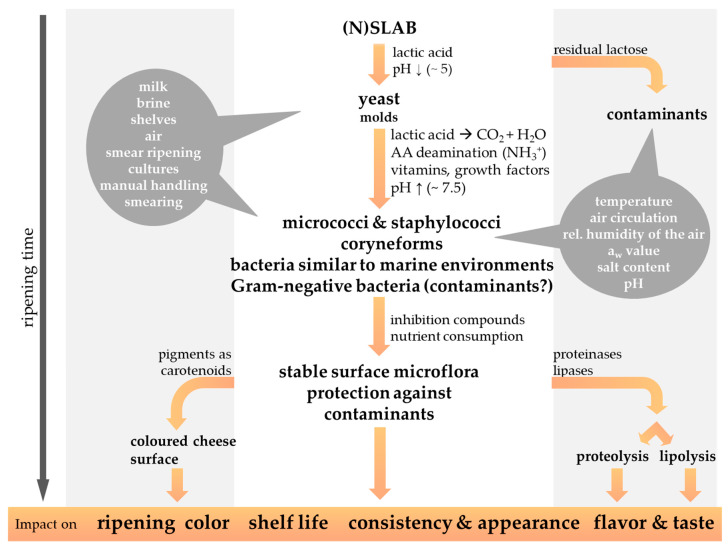
The microbial succession of the cheese surface microbiota during the ripening period contributes to the characteristics of smear-ripened cheese varieties. The possible origin of the smear microorganisms, as well as the physicochemical properties influencing the cheese smear development, are highlighted by a grey background. Gram-negative bacteria are regularly isolated from the surface of red-smear cheeses, but their significance as beneficial or contaminating bacteria is still under discussion (modified from Winkler et al., 2008 [48]).

The ripening of smear-ripened cheeses (Figure 2) is characterized by the development of a typical smear microbiota that contributes to a strong flavor and high levels of proteolysis and lipolysis mainly at the cheese surface [4,11]. Environmental parameters such as the ripening temperature, relative humidity, ripening time, the composition of the ‘house microbiota’, and the frequency and type of cheese curing direct the cheese ripening process [11].

Pressed and brined cheese curds are allowed to mature in a cheese aging cellar at controlled environmental climate conditions. Selected ripening temperatures comprise 8 to 20 °C for smear-ripened cheese [11,16,22]. Softer smear-ripened cheese varieties such as Münster or Limburger were ripened at a higher temperature range (15–20 °C), whereas harder varieties such as Trappist or Tilsit-like cheese ripen at a lower temperature range (12–15 °C) [11,16,22]. A high temperature of 20 °C to as high as >30 °C might be applied for one day as a warm pre-ripening step at the beginning of the ripening period [16]. The ripening temperature largely affects the growth rate of the microorganisms and their enzyme activities, whereby a higher temperature accelerates the ripening process. At an appropriate ripening temperature, a high relative humidity and exposition to an aerobic atmosphere a smear layer develops naturally on the cheese surface [24,32]. The humidity in the cheese’s surrounding environment ought to reach at least 95% relative humidity to prevent the cheese from drying out [16,22,24,32]. To strengthen the rind, smear-ripened cheese might be stored during a short period in a cheese aging room with a lower humidity [22]. Any excessive ventilation should be avoided during cheese ripening, but climate conditions need to be oxygenated for the growth of the mainly aerobic smear microbiota [16,24,32]. Ripening under anaerobic conditions can cause putrefaction of the cheese rind [22]. As mold may grow at aerobic conditions, which show a broad range in pH tolerance and feature xerotolerant properties in relation to the moist and salty properties of a cheese, there is an increased risk of mold growth on the cheese surface during the first days of cheese ripening [16]. To initiate a direct competition between mold and other microorganisms that leads to an outcompeting of mold due to their slower growth rates, the micro-habitat of the cheese surface is regularly disrupted during the ripening process, by washing the cheese as a curing technique, also termed smearing of cheese [11]. For large firm cheese varieties, the cheese surface is periodically brushed with a smear liquid using circular brushes, originally manually by the cheese maker but nowadays by the rotating equipment of a smearing machine [11,16,17,22]. The liquid used for smearing is composed either of saltwater (2–3% sodium chloride) or a whey liquid solution [16,17,22,32]. As discussed previously, the smear microbiota from mature cheese is usually no longer transferred from old to young (green) cheeses by the ‘old-young’ smearing technique due to safety reasons. Therefore, commercially available surface ripening starter cultures may be used to inoculate the smearing liquid [16,17,24,45]. Surface starter cultures may comprise individual strains or mixed starter cultures of *Debaryomyces hansenii*, *Geotrichum candidum*, *Brevibacterium linens*, *Brevibacterium casei*, *Staphylococcus equorum*, *Microbacterium gubbeenense*, *Glutamicibacter* spp., or *Corynebacterium casei* [11,16,17]. However, this limited diversity of strains does not reflect the high microbial diversity that naturally develops on the cheese surface during ripening [11,17]. Furthermore, it was demonstrated that although harbored in high numbers in the smearing liquid, individual strains might not be established on the cheese surface during the ripening process. This was observed for *Brevibacterium linens* on a French soft, red-smear cheese and for *Brevibacterium aurantiacum* and *Staphylococcus xylosus* strains, inoculated on the surface of Époisses cheese, which were outcompeted by adventitious strains from other species [45,46]. Neither a commercial smear starter culture (composed of *Debaryomyces hansenii*, *Galactomyces geotrichum*, *Glutamicibacter arilaitensis,* and *Brevibacterium aurantiacum*) inoculated into the cheese milk during processing was able to compete with the resident adventitious smear microbiota [34]. In contrast, Bockelmann and Hoppe-Seyler (2001) demonstrated the successful application of a defined surface-ripening starter culture composed of *Debaryomyces hansenii*, *Brevibacterium linens*, *Glutamicibacter nicotianae*, *Corynebacterium ammoniagenes,* and *Staphylococcus sciuri* for the ripening of Tilsit cheese [17]. Further, Zhao and colleagues (2022) observed a positive effect on the cheese surface smear when applying high-cell density cultures of *Brevibacterium linens* in acid whey to develop a deep orange surface smear within a few days, thus reducing the ripening period [35]. Nevertheless, recent studies monitoring the origin of the adventitious smear microbiota members revealed the ‘house microbiota’ as the major source for the smear microorganisms detected on European and American smear-ripened cheese varieties [12,13,47].

The smearing of the cheese is performed on an almost daily basis at the beginning of the ripening period and less frequently at a later stage. As the proper development of the smear microbiota is affected by the oxygen content in the surrounding environment, cheese wheels are frequently turned, to guarantee aerobic conditions all over the whole cheese surface [11]. Instead of smearing, smaller sized cheeses are rather high-pressure sprayed with a smear liquid [16,32]. Applying too much salt can cause the surface smear to develop a whitish coloration, whereas desiccation of the cheese surface indicates under-salted conditions [22]. A few days after the first smearing procedure non-uniformly distributed light yellow colored bloom appear on the surface of smear-ripened cheese. By periodical smearing, smear microorganisms are more and more equally and rapidly spread, and after 2–3 weeks ripening the desired smear microbiota develops and ensures uniform ripening of the cheese [11].

The smear microbiota covers the cheese surface and protects from the growth of microbial contaminants or desiccation of cheese [11,32]. The surface-smear microbiota exerts a hurdle effect against undesirable microorganisms that is provided by different modes of action, including competition (e.g., for essential nutrients and/or space) and amensalism (e.g., production of antimicrobial substances) [49]. Known as cheese spoilers are mainly mold, which are potentially capable of mycotoxin production, whereas inter alia *Listeria monocytogenes*, *Staphylococcus aureus*, *Salmonella* spp., *Brucella* spp. or Shiga Toxin-producing *Escherichia coli* (STEC) such as *E. coli* 0157:H7 represent important food-borne human pathogens of cheese [9,11,16,25,26,50,51,52,53]. Furthermore, the cheese flavor and taste, consistency and appearance, as well as the cheese shelf-life, are affected by the ripening process, as discussed in the following chapters. The production of pigments by the surface smear microbiota during further ripening of cheese render the cheese surface red-orange colored [11,16,22,54,55]. The duration of the ripening period differs in relation to the intensity of the cheese flavor and aroma appreciated, and according to the cheese matrix consistency and the intended cheese variety [11,22]. Soft and semi-soft cheese varieties might already be processed further after 2–3 weeks. The smear is then removed, and the cheese is wrapped or coated with protective layers before further maturation at lower temperatures, e.g., 10 °C [11,22]. In general, harder smear-ripened cheese varieties are ripened for longer periods, such as 1–6 months for semi-hard Tilsit-type cheese or 6–12 months for hard Gruyère-like cheeses [12].

## 4. The Microbiology of Smear-Ripened Cheese

Cheese is the habitat for two different communities of microbes. The first is found inside the cheese, containing primarily lactic acid bacteria, and the second is found on the surface of the cheese, where a diverse collection of microbes make up the rind [56]. Consequently, there exists a huge diversity after ripening on the cheese surface and in the cheese’s internal body [9]. Whereas microbial and biochemical processes in the cheese interior mainly influence the characteristics of mature internal bacterial ripened cheese types, so does the smear microbiota for surface-ripened cheese types [10]. However, internal and external cheese ripening is present for all kinds of cheese, but processes might differ to a lesser extent for cheese ripened in a coating or in plastic film, where physicochemical characteristics of cheese are equalized [9,10]. Whereas the cheese interior matrix, covered by a firm cheese rind, does not promote the growth of obligate aerobic microorganisms due to limited oxygen availability, the cheese surface is exposed to a highly oxygenic environment and will get covered by a mainly aerobic surface microbiota [9]. Consequently, physicochemical parameters differ for the cheese interior and exterior and thus determine the appearance and establishment of an individual differing microbiota on the cheese surface and in the cheese matrix.

Cheese harbors a complex microbial biocenosis in the interior and on the exterior. Thus, the analysis of its origin, composition, succession, and microbial interaction is a challenging and time-consuming task with need in high laboratory material requirements. Most of our knowledge about the typical cheese surface microorganisms is derived from culture-based techniques for the analysis of the cheese surface microbiota, which have been widely applied for decades [57,58,59,60]. Even though special cultivation media as e.g., milk agar imitating the nutrient conditions of green cheeses, modified milk agar simulating the nutritional properties of ripened cheeses as well as cultivation media supplemented with high salt concentrations comparable to the concentrations found on the cheese surface, or selective cultivation media were developed, at least 20–50% of the active microbial community may not be cultivable in vitro, even when analyzing simple fermented food matrices [17,34,58,61,62,63,64]. Apart from the failure to characterize stressed or weakened cells or minor populations, which are outcompeted in vitro by numerically more abundant microbial species, culture-based methodologies may not be an appropriate choice for the monitoring of the dynamics of complex microbial communities because they may lead to a misinterpretation of the actual microbial diversity [57,58,65]. The recent development of molecular analysis tools for the study of the cheese surface microbiota provides less time consuming, much more sensitive, specific, and accurate analysis methods, compared to the traditional culture-based methods [58,59]. Thus, recently developed molecular methodologies such as high-throughput sequencing opened a new dimension for further studies of one of the most complex and fascinating ecosystems in foods.

### 4.1. The Microbiota of the Cheese Interior

The milk applied for cheese production contains indigenous microorganisms, which may contribute to cheese milk fermentation and cheese ripening. Milk can be colonized during milking by microorganisms present on teat skin, milking equipment, water, animal feed, grass, soil, air, and further farm-related environments [6,66,67,68]. After milking, the milk contains a highly diverse mixture of bacteria with lactic acid bacteria such as *Lactococcus*, *Lactobacillus*, *Leuconostoc*, *Streptococcus,* and *Enterococcus* as the most abundant representatives. They derive mainly from ecological niches in the farm environment like plant materials, silage, cowsheds, teat surfaces, or animal feces [6]. Another major fraction in the milk is psychrotrophic bacteria such as *Acinetobacter*, *Pseudomonas*, or certain members of enterobacteria, which might proliferate during refrigerated storage of milk. Furthermore, Gram-positive bacteria other than lactic acid bacteria, such as *Propionibacterium*, *Staphylococcus*, *Corynebacterium*, *Brevibacterium,* as well as yeast and mold, are regularly harbored by raw milk [6,59,67]. Typically, individual bacteria genera occur in the range of 10^1^ to 10^4^ cfu ml^−1^ in milk [6]. The final microbial diversity in milk can differ from one farm to another depending on the animal feed (indoors or outdoors), the location of animals, and the lactation stage as shown in studies [69,70]. Previous studies also revealed microbiome changes in the cheese interior during the cheese ripening [66].

Adventitious microorganisms in the milk can positively impact the organoleptic properties of processed dairy products such as cheese and influence their sensory and textural properties [6]. However, appropriate handling of the raw milk prior to cheese production is crucial for the microbial quality of the cheese milk, as psychrophilic bacteria might flourish during cold storage and may even dominate dairy tank milk [6,40]. The psychrotrophic and psychrotolerant microbiota of raw milk is predominantly composed of Gram-negative bacteria (e.g., *Alcaligenes*, *Chromobacterium*, *Enterobacter*, *Flavobacterium*, *Pseudomonas* and *Serratia*), as well as Gram-positive bacteria (*Bacillus*, *Glutamicibacter*, *Clostridium*, *Lactobacillus*, *Listeria*, *Staphylococcus*, *Corynebacterium*, *Microbacterium* and *Micrococcus*) [9,71]. After cold storage of raw milk, Gram-negative bacteria can predominate and comprise up to 90% of the raw milk microbiota, which has been previously described in an American study [71,72]. During bacterial growth, extensively produced extracellular lipases and proteases might spoil dairy products and even lead to cheese defects [6,73,74]. Furthermore, raw milk can be contaminated by a variety of pathogens like *Campylobacter* spp., *Listeria monocytogenes*, *Salmonella* spp., or pathogenic *Escherichia coli* such as Shiga-toxin producing *E. coli* (STEC), or *Yersinia enterocolitica*, which are clearly associated with human illness and disease. Several well-documented milk-borne disease outbreaks during the last decades could be traced back to the consumption of raw, unpasteurized milk or the cheese made thereof [75,76].

For this reason, as well as due to safety reasons considering vegetative pathogens such as *Listeria monocytogenes*, pasteurization of milk is performed prior to cheese making for most cheese varieties, whereby approx. 99.9% of the indigenous bacteria in raw milk are depleted and milk enzymes such as proteases and lipases are denatured [40,77]. In fact, spores of *Bacillus* or *Clostridium*, some psychrotrophic, as well as thermoduric microorganisms, including species of *Glutamicibacter*, *Corynebacterium*, *Enterococcus*, *Micrococcus*, *Microbacterium,* and *Streptococcus* are able to survive the pasteurization procedure to some extent [9,77]. Nevertheless, in the year 1997, approximately 10% of the total cheese production concerned cheeses manufactured from raw milk in the European Union and Switzerland [78]. As raw milk cheeses ripen faster, acquire more rich and intense flavors and an increasing interest in traditional cheese products persists by consumers, there is still a trend for raw milk cheeses [38,40,78,79,80]. Interestingly, an abundant microbial biodiversity in cheese is not only thought to cause diverse and distinct sensory benefits. It may also protect against the establishment of undesirable microorganisms due to the presence of strains that provide an antagonistic effect to certain pathogens such as *Salmonella*, *Listeria monocytogenes*, Shiga Toxin-producing *Escherichia coli* (STEC), or *Staphylococcus aureus* [31,40,81]. Such an antagonistic effect has been attributed to the production of bacteriocins, bacteriocin-like substances or organic acids and the competition for nutrients [31,81]. It was recently shown that cheeses produced from raw milk showed significantly lower growth of *Listeria monocytogenes* than pasteurized-milk cheeses, possibly due to an increase in microbial competition [25]. However, although growth of *Listeria monocytogenes* in smear-ripened cheeses was negatively correlated with the relative abundance of two *Lactococcus* spp., the presence and relative abundance of *Streptococcus thermophilus* revealed a positive correlation with the growth of *Listeria monocytogenes* [25]. This observation demonstrates the complexity of the influence that the cheese starter microbiota exerts on the food safety in smear-ripened cheeses. The role that lactic acid bacteria and other members of the natural microbial communities found in milk and dairy products play in the defense against pathogenic microorganisms was recently reviewed in detail by Pepi and Focardi (2022) [7]. Additional benefits attributed to raw milk cheese refer to the positive impact of large quantities of lactic acid bacteria on the enteric human microbiota, possibly beneficial milk components protecting humans from asthma and allergies by so far unknown mechanisms and the balance of unsaturated fatty acids, which are beneficial to human health [40].

Whereas cheeses produced from raw milk might be extensively colonized by adventitious microorganisms, cheese manufactured from pasteurized milk can get recontaminated by secondary microbial contaminants during the cheese production process [71,82]. Bacteria in milk or rinsing water are able to form biofilms in dairy pipelines by adherence and aggregation on the stainless steel surfaces [71]. Microbial analysis of milk from output pipelines demonstrated an increase in microbial numbers after contact with pipelines containing a dairy biofilm [82]. Wooden vats covered with a microbial biofilm can also act as a source for the inoculation of pasteurized milk with a ‘starter factory’ for cheese production [83].

Biofilm formation in milk production and processing environments occurs in four discrete stages. First, organic milk compounds form a conditioning layer, which alters the physicochemical properties of the surface where the biofilm will be formed [71]. Second, single cells transported to the surface attach to the substratum and form bridges between single bacterial cells, mediated by fimbriae, pili, flagella, and bacterial extracellular polymeric substances (EPS). During the third and fourth stage, bacteria proliferate and the biofilm expands. Biofilm-forming bacteria in the dairy environment comprise mostly Gram-negative bacteria such as members of the genera *Proteus*, *Enterobacter*, *Citrobacter*, *Shigella*, *Escherichia*, *Edwardsiella*, *Aeromonas*, *Plesiomonas*, *Moraxella*, *Alcaligenes* and *Pseudomonas* or Gram-positive bacteria such as *Staphylococcus*, *Bacillus*, *Listeria* and lactic acid bacteria (*Streptococcus*, *Leuconostoc*, *Pediococcus*) [71]. The genera *Pseudomonas* and *Streptococcus* represent the bacteria which are most frequently isolated from the surfaces of food processing areas in the food industry [71]. Biofilms harboring multi-species communities predominate in nature, but sections with elevated temperatures in the dairy plant environment are known to select for mono-species biofilms, for example, thermophilic bacilli [71,84,85]. Furthermore, biofilms reveal an increased resistance to chemical disinfection and may promote the survival of human pathogens and food-spoiling bacteria in hostile environments [71,84,85]. Cleaning procedures used in the dairy environment need to be evaluated and have to be applied in an accurate way, as the cleaning of complex processing equipment is difficult and standard cleaning procedures often may not adequately remove all dairy biofilms [71]. Concerning foodborne pathogens related to cheese production, *Listeria monocytogenes* was confirmed to survive in biofilms originally produced by *Pseudomonas* species. Cells of *Pseudomonas fragi* or *Flavobacterium* spp. were identified as primary colonizers on surfaces, which pioneer later adhesion of *Listeria monocytogenes* [71]. In the past, different milk and cheese-related environmental surfaces were confirmed as a source for recontamination of cheese with *Listeria monocytogenes* [30,86].

Whether microorganisms are already present in raw milk as natural contaminants, secondary contaminants or artificially added as defined starter cultures to the cheese milk before curdling, the skilled cheese maker knows how to control growth and metabolism of the starter microorganisms to ensure a high cheese quality and safety [9]. When appropriately attended and controlled, properly selected lactic acid bacteria (e.g., *Lactococcus lactis*, *Lactobacillus delbrueckii* subsp. *lactis, L. delbrueckii* subsp. *bulgaricus*, *L. helveticus*, *Leuconostoc* spp., *Streptococcus* spp.) were used as primary starter cultures for the fermentation of lactose. They promote the production of high-quality cheese that develops the desired flavor and the appreciated color and texture [9,87]. The above-mentioned starter bacteria are predominant at the beginning of the ripening process, as they grow to ≥10^8^ cfu g^−1^ in cheese within hours [77]. They are mainly involved in the production of lactic acid from lactose, and many of them also produce volatile flavor compounds, CO_2,_ and citrate and thus contribute to the open texture of some cheese types. Furthermore, they contribute to a low pH and E_h_, and produce antimicrobial compounds against spoilage and pathogenic microorganisms, which supports the microbial safety of cheese [87]. Various of these starter bacteria synthesize intracellular enzymes, which are released into the cheese environment when the bacteria lose their viability and autolyze [77,87].

Secondary starter cultures applied during cheese production comprise non-starter lactic acid bacteria (NSLAB), propionibacteria, coryneform bacteria, staphylococci, yeast, and mold. They are intended to further contribute to the organoleptic and textural properties of the cheese. For instance, cultures of *Propionibacterium freudenreichii* are applied in Swiss-type cheese to trigger the formation of large eyes in the cheese by fermentation of lactate to CO_2_, whereas the produced propionic and acetic acid contributes to the characteristic flavor development of this cheese variety [77]. Secondary starter cultures may originate from raw milk and factory environments or may be added as adjunct cultures for the control and acceleration of cheese ripening [9,16,87]. They can be inoculated into the cheese milk during production or may be applied as surface starter cultures by adding them to the smearing liquid during the cheese ripening, where their microbial activity on the cheese surface is much appreciated [9,16,77]. However, when cheese milk is inoculated with smear starter cultures, these microorganisms might rather be located inside the cheese body than on the surface [34].

At the end of the cheese ripening period, the cheese interior is mainly colonized by bacteria from the starter culture, which represent mainly the genera *Lactococcus*, *Lactobacillus,* and *Streptococcus* [88]. The abundance of the genera *Lactococcus*, *Lactobacillus,* and *Streptococcus* in the core bacterial community of ripened cheese was also confirmed by more recent studies applying culture-independent techniques, such as high-throughput sequencing. Interestingly, the culture-independent analyses of the microbial diversity and dynamics in different French cheese varieties, Italian Plaisentif cheese or Danish Danbo cheese confirmed the results from culture-dependent plating results with only minor exceptions [89,90,91]. However, the culture-independent analyses did not only reveal microorganisms that were already expected to be part of the cheese interior, such as *Glutamicibacter*, *Bifidobacterium*, *Brachybacterium*, *Brevibacterium*, *Clostridium*, *Enterococcus*, *Halomonas*, *Lactobacillus*, *Lactococcus*, *Leuconostoc*, *Pediococcus*, *Penicillium*, *Pseudoalteromonas*, *Pseudomonas*, *Psychrobacter*, *Staphylococcus*, *Streptococcus* and *Vibrio.* Their application also identified bacteria that were not previously associated with artisanal soft, semi-hard, and hard cheese, such as representatives of the genera Faecalibacterium, Helcococcus, or *Prevotella* [92]. Other genera were detected for the first time in the core of cheese, such as *Glutamicibacter* and *Brachybacterium* in goat cheese. These genera are commonly detected on the cheese surface but were so far not known as part of the core microflora of goat cheese. Furthermore, *Pseudoalteromonas* spp., which are usually regarded as marine bacteria, have previously been detected on the surface of smear-ripened cheese, but their presence in the interior of soft and semihard cow milk cheeses was an unexpected finding [92].

### 4.2. The Smear Microbiota on the Cheese Surface

After molding and pressing of cheese prior to the ripening, cheese curds are uniform, and the microorganisms acquired during the cheese production process, in general, are distributed in the interior and exterior of the cheese. Differing physicochemical properties and the smearing technique applied to the cheese surface lead to a selective pressure favoring the development of a complex red smear microbiota [32]. The mainly aerobic bacteria of the smear microbiota are able to catabolize more effectively important energy sources, such as lipids, amino acids, or lactic acid available at the cheese surface, than the fermentative lactic acid bacteria, which are better adapted to the inner, anaerobic part of the cheese wheel [93].

Besides originating from potential starter cultures and cheese milk, naturally occurring indigenous smear microorganisms derive from the brine that is used as smearing liquid or the skin of arms and hands of cheese workers, as well as from the wooden shelves, the air, and the environment of the ripening cellars, which in total represents the ‘house microbiota’ of a cheese dairy [12,13,14,94,95]. This ‘house microbiota’ is dairy facility-specific and does not seem to be affected by seasonal variations, as suggested by American and French studies analyzing the microbiota in cheesemaking plants producing either pasteurized or raw milk cheese [12,94]. Interestingly, functional habitats are colonized by similar communities of microorganisms occupying the same surface types, whereby the cheese making environment reflects the microbial community detected on smear-ripened cheese surfaces [12,13]. During the maturation of cheese, the surfaces are mainly colonized by halotolerant Gamma-proteobacteria such as *Pseudoalteromonas*, *Psychrobacter, Halomonas*, and *Vibrio*, as well as Gram-positive *Brevibacterium*, *Corynebacterium*, *Micrococcus,* and *Staphylococcus* species. Fungal communities such as *Debaryomyces* or *Penicillium commune* were detected throughout American artisan cheese making plants [12]. Wooden shelves used for the ripening of cheeses may represent another important source. Cleaning processes do not remove all organisms from the wooden shelves, as microorganisms are detected on wooden fibers and in cracks of wooden shelves after cleaning, with total colony counts as high as 10^5^ cfu cm^−2^ [13,14,95]. In particular, members of *Micrococcus*, *Corynebacterium*, *Staphylococcus*, *Debaryomyces,* and *Geotrichum* were found to be dominant on wooden shelves [13,94]. The use of stainless steel as an alternative can improve hygienic standards, as microorganisms, in general, are less abundant on this material, but a steel surface diminishes the drying of the cheese rind [13]. Furthermore, brine and smearing liquid might be extensively colonized with up to 10^4^ cfu mL^−1^ of various microorganisms such as *Acinetobacter*, *Brevibacterium*, *Brevundimonas*, *Corynebacterium*, *Flavobacterium*, *Halomonas, Lactobacillus*, *Lactococcus*, *Pseudoalteromonas*, *Pseudomonas*, *Psychrobacter*, and *Staphylococcus*, which originate from brine, tap water or sea salt [12,13,96,97,98]. Nowadays, cheese is treated manually to a minor degree. Consequently, typical smear microorganisms like *Agrococcus*, *Glutamicibacter*, *Brevibacterium*, *Corynebacterium*, *Debaryomyces*, *Microbacterium*, *Psychrobacter,* and *Staphylococcus* are less often transferred via the smearing procedure [13]. This is, in particular, the case for bacteria deriving from human skin. Recent microbiome analyses using culture-independent, high-throughput sequencing techniques to track the origin of cheese microorganisms provided a further in-depth view on the microbial sources for various cheese types [14,99]. Using a source tracker tool, Sun and colleagues (2021) showed for traditional cheese production bidirectional interactions between the milking environment and the microbial composition of milk, the microbial communities in dairy products and the cheese-making tools, as well as in ripening cellars and microbial communities on cheese rind. This observation provides further evidence that important taxa from wooden vat surfaces may be transferred to the raw milk and are able to persist during cheese ripening and may even dominate the end-product [99]. Using a 16S rRNA gene pyrosequencing approach for the investigation of Belgian Herve cheese, Delcenserie and colleagues identified a specific microbiota present in both the raw milk cheese and in the pasteurized milk cheese. Only a small part of the microbiota was found to be unique for the raw milk or the pasteurized milk cheese [100].

**Figure 3:**
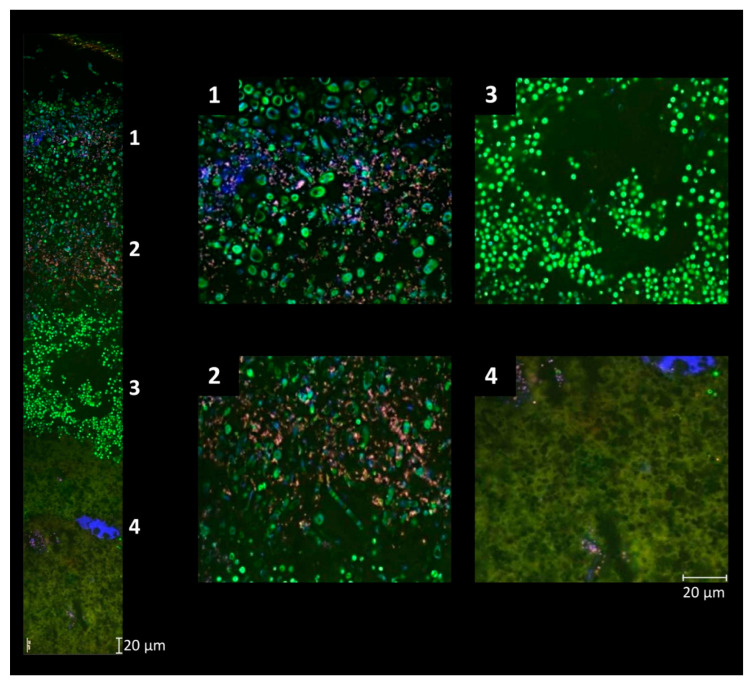
Simultaneous visualization of eukaryotic cells (green) and bacterial cells (red-pink) by Fluorescence in-situ Hybridization (FISH) analysis of a vertical section from the surface of a smear-ripened cheese. Yeast cells are detected in particular at the bottom level of the smear microbiota (3), as these microorganisms colonize the cheese surface during the early stages of the cheese ripening period. In later ripening stages, dominant bacterial cells overgrow the initial layer of yeast and predominate the top layers of the smear microbiota (1 & 2). Bacterial cells originating from the cheese starter culture can be detected in patches in the cheese matrix (4) (Image with kind permission from Annette Moter; Biofilmcenter, Department of Microbiology, Infectious Diseases and Immunology, Charité, Universitätsmedizin Berlin, Germany).

The ripening process itself is characterized by the successive development of a smear microbiota on the surface of red-smear cheese [13,101,102]. Young cheeses feature a low pH (e.g., pH 5) on the curd, caused by acidification due to lactic acid bacteria, whereby the brining of the cheese as a final manufacturing step lowers the cheese moisture content [9,11,32,103]. During the initial stage of ripening acid- and salt-tolerant yeast dominate the cheese surface (Figure 3).

Yeast species such as *Candida catenulata*, *Debaryomyces hansenii*, *Geotrichum candidum* and *Yarrowia lipolytica* grow with an average abundance of about 10^4^ cfu cm^−2^, deacidify the cheese surface by degrading the lactate produced by the starter culture bacteria [9,11,17,24,32,63,104,105,106]. It is known that yeast also contribute to the production of volatile compounds [107]. Yeast growth prevents the cheese surface from drying out and their proteolytic and lipolytic activities contribute to the cheese ripening process [11]. Using gas chromatography-mass spectrometry to detect volatile compounds of the cheese surface and correlation analysis, it was possible to associate *D. hansenii* with the production of alcohols and carboxylic acids and *G. candidum* with the production of sulfur compounds [23]. Furthermore, yeast deaminate amino acids to ketoacids and ammonia, they lipolyse lipids and stimulate the increase in smear bacteria by producing growth-supporting molecules such as vitamins, pantothenic acid, niacin, or riboflavin [11,32,105]. Further important flavor compounds (volatile acids, carbonyl compounds) formed by yeast are fruity ester flavors (e.g., *Kluyveromyces marxianus*) or sulfur flavors (e.g., *Candida krusei*) [16]. By releasing alkaline metabolites pH on the surface of semi-soft smear cheese increases to >6.5 within 2–4 days of initial ripening. Thus, yeast generate a convenient habitat for typical acid sensitive red smear bacteria [11,32]. Mounier (2015) describes commensalism between yeast and bacteria as the most documented interaction occurring in smear cheese [108]. The knowledge about this interplay was also used in a study, where an artificial consortium of smear bacteria and yeast was applied on the surface of young Cheddar cheese curd to modify the flavor and appearance of Cheddar cheese [109].

Staphylococci, which are able to grow at low pH values, can also predominate early stages of cheese ripening [9,110]. On German Tilsit cheese, staphylococci represent the dominant bacterial portion of the smear microbiota in the first two weeks of the ripening period and decreased thereafter drastically [102]. Experimental cheese manufacturing trials demonstrated the importance of yeast and staphylococci for mold inhibition on cheese surfaces [32]. Consequently, a variety of *Staphylococcus* species, such as *S. equorum*, *S. xylosus*, *S. warneri* or *S. pasteuri*, were regularly isolated from various smear-ripened cheeses produced either from raw milk or pasteurized milk in European countries such as Germany, France, Italy, Switzerland or Ireland [11,17,32,33,63,102,110,111,112,113,114,115]. Culture-based analysis of the cheese smear microbiota of five different European smear-ripened cheese varieties revealed that 14% of the isolates belonged to the genus *Staphylococcus*, which was also found to be the most prominent genus on Gubbeen cheese [9]. *Micrococcus* species isolated from the cheese surface microbiota of European smear-ripened cheese belonged mainly to *M. luteus,* or *M. lylae* and accounted for 0.1–20% of the bacterial counts on the surface of Chaumes cheese [17,32,33,63,111]. By their production of extracellular proteolytic and lipolytic enzymes, staphylococci contribute to the typical aroma and flavor development and promote the establishment of coryneform bacteria by production of growth factors, which is important for the typical succession of microbes on the cheese surface during ripening of the cheese [33]. In general, staphylococci are later replaced by coryneform bacteria, which dominate the later stages of the ripening period and represent 50–95% of the total cultivable smear microflora on mature German Tilsit-type cheese or Gubbeen cheese [32,33]. On the surface of German Tilsit cheese, *Corynebacterium* spp. was found to be present at low levels during the first two weeks of ripening; its levels increased thereafter until they reached a maximum at week 8 of the ripening period [102]. Coryneform bacteria prefer the salty and pH-neutral cheese surface and contribute considerably to the typical cheese flavor and appearance [116]. Furthermore, carotenoid production by coryneform bacteria renders the cheese surface color crème, yellow to red [16,17,117]. Although recent molecular taxonomic analysis revealed that the coryneform bacteria do not designate a phylogenetic taxon, this designation is still widely used to group genera such as *Glutamicibacter*, *Brevibacterium*, *Corynebacterium* or *Microbacterium*, which share a similar coryneform cell morphology [11].

From the beginning of the investigation of the cheese smear microbiota, brevibacteria, mainly *B. linens*, were considered the predominant bacteria in cheese smear due to their ubiquitous presence on different cheese varieties and their enzymatic and biochemical characteristics that strongly influence the development of the typical aroma and texture during the ripening process [11,17,22,32,62,116,118]. Further brevibacteria isolated from cheese surfaces comprise *Brevibacterium aurantiacum*, *Brevibacterium casei,* and *Brevibacterium epidermidis* [119,120]. An intense production of extracellular and intracellular proteinases has been reported for these bacteria, which contribute to the production of aromatic sulfur compounds, such as methanethiol, hydrogen sulfide, dimethylsulfide, S-methylthioacetate, 4-trithiapentane, and ethional, which are typical for smear-ripened cheeses [16,17,23]. Moreover, certain strains of *Brevibacterium linens* have been reported to produce different antifungal and antilisterial bacteriocins that contribute to pathogen and spoilage prevention [11,16,35,117,121]. In general, *Brevibacterium linens* were found to be essential for color development on red smear cheese, as it is known for its bright red-orange carotenoid type pigments [11,16,17,35,117,121], although a direct contribution to cheese coloration seems only be likely for a number higher than 10^9^ cfu cm^−2^ [16,17,117]. The cheese smear microbiota of German Tilsit cheese comprised 1–15% *Brevibacterium linens* in culture-based analyses and rarely up to 30% in the smear microbiota of other cheese varieties [11,16,117]. On mature cheese surfaces, brevibacteria generally contribute to less than 5% of the total bacterial counts [119]. Due to their interesting technological properties, selected strains of *Brevibacterium linens* are often applied as surface smear starter cultures. Nevertheless, recent studies revealed that *Brevibacterium linens* might not be established successfully on various surface-ripened cheese varieties, and its major contribution to the cheese characteristics seems to be overestimated [33,61,110,116,117,122]. It was previously shown that certain members of *Staphylococcus* and coryneform bacteria may inhibit the growth of *Brevibacterium linens* by bacteriocin production at the beginning of the ripening process [62].

*Arthrobacter* species usually found in the surface smear of red-smear cheese like *A. nicotianae*, *A. arilaitensis,* and *A. bergerei* have been recently assigned to the novel genus *Glutamicibacter*, as *Glutamicibacter arilaitensis*, *Glutamicibacter bergerei*, and *Glutamicibacter nicotianae* [123]. They usually appear at the early to medium stages of the ripening process and are more or less abundant in the surface smear [11,17,32,62,63,111,116,124,125]. Many strains are in particular of high importance for the typical coloration of the cheese surface, as they produce yellow pigments, which change to red-brown color under the influence of proteolytic bacteria like *Brevibacterium linens* [16,17,55,61,126,127]. Yellow pigments concern carotenoids produced in response to light-induction, growth temperature, or salt concentration. Further, carotenoids in *Arthrobacter* and *Glutamicibacter* might function as membrane-integrated antioxidants that protect the cells from oxidation and stress or stabilize the cell membrane at low temperatures [127,128]. The red pigments of *Glutamicibacter arilaitensis* have been characterized and identified in a recently conducted study [55]. The production of bacteriocins, e.g., against *Listeria monocytogenes*, is also described for individual strains of *Arthrobacter* and *Glutamicibacter*. However, *Brevibacterium*, *Arthrobacter* and *Glutamicibacter* are not the only smear bacteria that show antilisterial activity. A study on 299 isolates obtained from German smear cheeses, identified 30 strains that inhibited at least one strain of *Listeria monocytogenes* on solid medium. These strains comprised members of *Arthrobacter, Brevibacterium, Corynebacterium*, *Enterococcus, Micrococcus* and *Microbacterium* [129]. In line with these observations, a recent study by Falardeau and colleagues (2023) revealed that growth of *Listeria monocytogenes* in cheeses was negatively correlated with the relative abundances of *Brevibacterium aurantiacum* [25].

So far, only few studies considered the enzymatic characteristics of *Arthrobacter* and *Glutamicibacter* species in relation to cheese smear [11]. Recent sequencing of the whole genome of *Glutamicibacter arilaitensis* strains Re117 revealed that the genome encoded a bunch of proteins revealing putative protein degradation functions, lipase activities, carotenoid biosynthesis and further specificities linked to its adaption to the cheese surface habitat [125]. In the meantime, the occurrence of *Glutamicibacter arilaitensis* could be associated with alcohols, carboxylic acids and ketones produced on the surface of smear cheese [23]. Culturing of *Arthrobacter* and *Glutamicibacter* strains in a liquid milk model system revealed an atypical smell for smear-ripened cheese, which changed to a fruity Tilsit-cheese like flavor when growing in co-culture with *Brevibacterium linens* [16,17]. Other yellow pigmented coryneform bacteria found on the surface of smear-ripened cheese included *Brachybacterium* and *Microbacterium*, e.g., *M. gubbeenense* species [11,16,62,63,111,128]. Representatives of the genus *Microbacterium* tend to be isolated during later ripening stages in high numbers, as they are unable to grow at a pH below 5.8 [9,62]. These bacteria are able to produce methanethiol, a major flavor compound of smear-ripened cheeses [62]. For *Microbacterium gubbeenense* growth promoting effects on *Brevibacterium linens* and vice versa were described in literature [16].

Several *Corynebacterium* species such as *C. casei*, *C. variabile*, *C. mooreparkense* represent a major part of the cheese surface smear microbiota and establish in particular at later stages of ripening [11,32,33,62,63,111,116,130]. According to a study by Brennan and colleagues (2002) corynebacteria tolerate a low pH of 4.9 and 8% sodium chloride concentrations [62]. Due to their fast growth on the cheese surface they reach high numbers (>10^9^ cfu cm^−2^) and may comprise as much as 70% of total bacterial counts in the surface smear, thereby protecting the cheese surface from colonization by undesired microbial contaminants [16,32,131]. Their contribution to the cheese aroma seems not very pronounced, as they show only weak esterase, lipase and leucine arylamidase activities [11,16]. Recently, the whole genomes of two *Corynebacterium casei* strains (UCMA 3821 and DSM 44701^T^) were sequenced and provide valuable information for further studies investigating the functional properties of *C. casei*, such as aroma compound production, or the ability to grow on the cheese surface supported by iron siderophore transport components [93,132].

*Enterococcus* species, mainly *E. faecalis*, *E. faecium*, *E. italicus* and *E. durans* belonging to the non-starter lactic acid bacteria are usually detected at the beginning of the ripening period on the surface and in cheese [11,111,133,134,135] As they tolerate low acid and salt conditions, they are adapted to various fermented food systems. Mainly in cheese produced in the Mediterranean area, enterococci contribute to cheese ripening and aroma development by proteolysis, lipolysis and diacetyl production [134,135]. Furthermore, they are known to produce bacteriocins that negatively affect the growth of *Listeria monocytogenes*, *Staphylococcus aureus* or *Clostridium perfringens* [81,133,135]. However, according to the European Food Safety Authority (EFSA) enterococci do not belong to the bacteria that possess the qualified presumption of safety (QPS) status when intended as biological culture added to foods or feeds, because no taxonomic unit within the genus *Enterococcus* can be considered as free of pathogenic species [136,137]. Also, the Food and Drug Administration (FDA) in the United States of America did not consider enterococci as ‘generally recognized as safe’ (GRAS), due to their putative virulence factors and their status as emerging human pathogen [133,135]. Enterococci exhibit a high rate of intrinsic antibiotics tolerance and are carriers of antibiotic resistance genes. Thus, the cheese ecosystem may provide a potential reservoir for antibiotic resistance gene exchange between enterococci and other bacteria [135]. Furthermore, enterococci bear the risk of producing biogenic amines, which can lead to intoxication upon cheese consumption [138]. More than 90% of *Enterococcus faecium* strains isolated from different types of cheese showed the ability for tyramine production in vitro, and similar observations were made for strains of *Enterococcus faecalis* and *Enterococcus durans* [138,139]. In drinking water enterococci are considered as indicators of fecal contamination [133,135].

Gram-negative bacteria such as members of *Enterobacterales* were originally not expected to belong to the characteristic microflora of the cheese smear, because they usually do not tolerate low pH and high salt concentrations. They have been mostly regarded as unwanted contaminants and indicator organisms for low process hygiene [16,111,140,141,142]. However, more recent studies combining cultural, genomic and metagenomic approaches to investigate the occurrence of halophilic and halotolerant bacteria in cheese demonstrated that Gram-negative species are particularly abundant in cheeses with high moisture, such as washed-rind cheeses [18,46,143,144]. Moreover, a study by Irlinger and Monnet (2021) on the temporal differences in the microbial composition of Époisses cheese rinds during ripening and storage revealed that at the end of the ripening period among the 18 most abundant bacterial species detected, 14 were Gram-negative bacteria, mainly from the genera *Psychrobacter*, *Vibrio*, *Halomonas* and *Mesonia* [46]. In earlier studies Proteobacteria such as *Enterobacter*, *Klebsiella*, *Citrobacter*, *Escherichia*, *Pseudomonas*, *Halomonas*, *Psychrobacter*, *Pseudoalteromonas* and *Vibrio* were already detected as part of the surface microbiota of smear-ripened cheese [18,24,32,33,63,145]. Proteobacteria identified in cheese could be assigned to a variety of different families (and according genera), such as *Enterobacteriaceae* (*Citrobacter*, *Enterobacter*, *Escherichia*, *Hafnia*, *Klebsiella*, *Kluyvera*, *Morganella*, *Proteus*, *Providencia*, *Raoultella*, *Serratia*), *Halomonadaceae* (*Halomonas*), *Alcaligenaceae* (*Alcaligenes, Tetrathiobacter*), *Caulobacteriaceae* (*Brevundimonas*), *Moraxellaceae* (*Acinetobacter*, *Moraxella*, *Psychrobacter*), *Oceanospirillaceae* (*Marinomonas*), *Pseudoalteromonadaceae* (*Pseudoalteromonas*), *Pseudomonadaceae* (*Pseudomonas*), *Vibrionaceae* (*Vibrio*) and *Xanthomonadaceae* (*Stenotrophomonas*) [12,18,21,92,141,146,147,148]. On the surface of semi-hard smear-ripened cheeses numbers of Gram-negative bacteria can reach up to 10^5^ cfu cm^−2^ or cfu g^−1^ surface smear and isolates can account for 32% of the overall bacterial biodiversity on the cheese surface [16,18,24,32,146]. Though, on soft cheese varieties numbers for Gram-negative bacteria might even be higher, with up to 10^8^ cfu g^−1^ [16,24,32]. The higher pH, the higher water activity and the lower salt content on soft cheese varieties might diminish the decrease in Gram-negative microorganisms during ripening and thus lead to higher numbers [32]. Overall observed differences in colony counts of Proteobacteria on similar cheese types might arise from different production locations, or due to seasonal variability in raw milk composition. High numbers of *Enterobacteriaceae* at the beginning of the ripening period usually decrease over the ripening time, due to the combined effects of different physicochemical parameters [145].

Traditionally, the occurrence of Proteobacteria in the surface-smear of cheese was recognized as a hygiene related challenge as well as a food safety issue, as several detected species are attributed to opportunistic pathogenicity [141,149,150]. Moreover, various Gram-negative bacteria showing resistance to antibiotics were regularly isolated from different types of food, including cheese [151,152,153]. Furthermore, the production of high concentrations of biogenic amines by decarboxylation of amino acids is a well-known feature of members of Enterobacterales that may cause toxic effects in consumers [49,74,140,141,154,155,156]. Besides the negative impact of Proteobacteria on hygiene and safety aspects during cheese production, also the aesthetic properties may likewise be strongly affected. Coliform bacteria at high levels in cheese can affect the cheese quality by provoking early gas defects within the first days of manufacture, which may further result in a negative effect on the texture and the development of off-flavors [74].

Whereas some authors place emphasis on the negative aspects of Gram-negative bacteria in the cheese surface smear, others accentuate their positive contribution to the cheese ripening process [6,24,32,141,142,146,148,157,158,159]. Nevertheless, the discussion in the scientific community is still going on, whereby the main intention is to produce a safe food product of constant quality, to enhance the cheese sensory quality and to support the growth of desired ripening bacteria whilst preventing from spoilage microorganisms [16,17,24,122]. However, more recent studies suggest that *Proteus* and other Enterobacterales produce volatile organic flavor compounds that contribute to the organoleptic properties of surface-ripened cheese and that they are part of the typical house microbiota that shape the organoleptic properties of the cheese, rather than represent unwanted contaminants, which is discussed in more detail in a previous article from Ritschard et al. (2022) [18].

Besides Gram-negative bacteria that are typical representatives of marine environments (*Halomonas* and *Vibrio*), marine lactic acid bacteria which adapted to halophilic conditions such as *Marinilactibacillus psychrotolerans*, *Alkalibacterium kapii,* and *Alkalibacterium psychrotolerans* or *Facklamia tabacinasalis* are also frequently reported to be present in cheese surface smear [47,64,111,160,161]. Ishikawa and colleagues proposed sea salt added to cheese during ripening as origin of such marine bacteria, whereby the halophilic conditions and the low temperature promote a niche for the development of the bacteria that adapted to these harsh environmental conditions [160,162]. During earlier ripening stages marine lactic acid bacteria are supposed to inhibit growth of *Listeria monocytogenes* on the cheese surface [40,64,81,111,163]. However, according to a recent study that systematically characterized the halophilic and halotolerant bacteria in the rinds of 13 selected traditional cheeses via cultural, genomic and metagenomic methods, the predominant Gram-positive species were notably *Brevibacterium aurantiacum* and *Staphylococcus equorum*, which are also frequently added as surface starter cultures [143].

The recent application of state-of-the-art high-throughput sequencing analysis techniques for the analysis of cheese surface smear provided profound and novel insights into the compositions of the cheese surface microbiota [12,60,91,92]. Members of the genera *Flavobacterium*, *Tetragenococcus*, *Helcococcus*, *Prevotella*, *Pediococcus*, *Faecalibacterium*, *Brevundimonas*, *Nocardiopsis* or *Yaniella* were for the first time described as part of the cheese surface microbiota [12,60,92]. Consequently, scientific literature does not provide relevant information concerning the potential contribution of these bacteria to the cheese ripening process.

Growth of mold on the surface of non-mold ripened cheeses is usually not appreciated, due to putative mycotoxin production and presumably undesirable effects such as discoloration which have a negative impact on the quality and safety of the cheese. Nonetheless, microbial analysis of the cheese surface smear revealed the presence of a broad variety of mold such as *Aspergillus versicolor*, *Fusarium domesticum*, *Mucor plumbeus*, *Penicillium commune*, *Penicillium nalgiovense*, *Scropulariopsis brevicaulis*, *Trichothecium domesticum*, and *Trichothecium roseum* on the surface of smear-ripened cheese [12,60,164,165,166]. However, sometimes the growth of non-toxic mold is desirable because it reduces the stickiness of smeared cheese surfaces [164,167]. Microbial source tracking identified cow teats as a major source for fungi in raw milk and suggested a potential carryover from raw milk and other sources such as hands, milk buckets, wooden vats or the air to the cheesemaking rooms [99].

At the end of the ripening process, the smear is composed of an ordinarily stable microbiota influenced by the competition for nutrients, depletion and production of substrates and the local properties and conditions on the cheese (Table 2) [168]. A review by Mounier (2015) provides a general overview about the microbial interactions in smear-ripened cheeses, implying commensalism between yeast and bacteria, which results in production of stimulatory compounds, surface deacidification, competition for space and nutrients. Furthermore, amensalism as well as parasitism may occur in the surface-smear ecosystem, as it is known that competition for peptides and amino-acids or the harvesting of iron play an important role in the interaction between the cheese microbiota and pathogens [108]. As cheese is an iron-restricted medium, due to glycoproteins that are able to chelate iron and due to iron oxidation to its insoluble form Fe^3+^ at neutral pH, iron availability might be a limiting factor for the growth of typical cheese surface bacteria [108,169]. Typically, yeast cell counts reach highest numbers during the deacidification of the cheese surface in the first days of ripening with up to 10^7^ cfu cm^−2^, whereas smear bacteria usually rise thereafter and comprise 10^9^ to 10^10^ cfu cm^−2^ after approx. 12 days of ripening [9]. The highest bacterial population levels are commonly detected after rapid deacidification of the cheese surface by yeast [168].

Concerning the population dynamics, a succession of microorganisms was monitored on the surface of Gubbeen cheese, where staphylococci dominated earlier ripening stages, whereas coryneform bacteria predominated during later ripening stages. Such a microbial succession was less pronounced for Limburger, Livarot, Reblochon or Tilsit cheese [9]. According to Cogan and colleagues (2014) only little information is available whether changes in the microbial dynamics occur and whether a distinct progression of microorganisms happens [170]. Generally, the biodiversity on the cheese surface is usually increasing during ripening and as a general principle, more acid-sensitive bacteria seem to develop during later ripening stages [9,168]. These observations from culture-based investigations of the surface-smear microbiota received further support by culture-independent analyses that also demonstrated an increase in the relative abundance of rare taxa by the acquisition of microbes along the production process of smear cheese [99]. Monitoring of the succession of the microbial population during the commercial production and subsequent ripening of smear-ripened cheese by 16S rRNA gene sequencing revealed that the production and ripening of smear-ripened cheese could be divided into three stages, and that the microbiota composition of samples from the first week of production, the second week of production, and at supermarket shelf life all differed. The most significant changes in the composition of the microbiota occurred during the ripening process on the shelves. In particular, Gram-negative bacterial species of Proteobacteria, Campylobacteria, and Fusobacteria were identified, which may play an essential role in ripening and thus contribute to the organoleptic characteristics of the cheese [171]. But overall, the composition of the microbiome of cheese seems to be rather stable, as the results from a recent study investigating the microbiota in Quebec’s Terroir Cheese over two years using a metabarcoding approach suggested the persistence of the microbiota over the whole study period [144].

**Table 2:** Phylogenetic classification and characteristics of selected bacterial genera regularly detected on the surface of smear-ripened cheese.

Phylum Order	Family	Genus	Atmospheric Conditions	% of Smear Microbiota ^a, b^	Contribution to Cheese Ripening	References
**Firmicutes**						
Bacillales	*Staphylococcaceae*	***Staphylococcus***	facultative anaerobic	5–18% ^a,b^	proteases, lipases, growth factors, flavor compounds, bacteriocins	[23,32,33,47,60,62,65,91,100,143,144,161,172,173]
Lactobacillales	*Carnobacteriaceae*	***Alkalibacterium***	facultative anaerobic	not known	lactic acid	[64,91,161,172,173]
Lactobacillales	*Carnobacteriaceae*	***Marinilactibacillus***	facultative anaerobic	2–5% ^a,b^	lactic acid, flavor compounds	[64,91,100,160,162,171,172]
Lactobacillales	*Aerococcaceae*	***Facklamia***	facultative anaerobic	0.6% ^a^	lipases	[92,172,173]
Lactobacillales	*Lactobacillaceae*	***Lactobacillus***	anaerobic	0.2% ^a^	>proteases, lipases, lactic acid	[65,78,92,172,173]
Lactobacillales	*Enterococcaceae*	***Enterococcus***	facultative anaerobic	<1% ^a,b^	proteases, lipases, flavor compounds, bacteriocins	[32,81,100,133,134,135]
**Actinobacteria**						
Actinomycetales	*Micrococcaceae*	***Arthrobacter***	aerobic	0.1–5% ^a,b^	proteinases, lipases, flavor compounds, pigments, bacteriocins	[32,60,65,89,92,100,125,143,172]
Actinomycetales	*Micrococcaceae*	***Micrococcus***	aerobic	0.1–20% ^b^	proteinases, lipases, pigments	[32,143,174]
Actinomycetales	*Brevibacteriaceae*	***Brevibacterium***	aerobic	1–30% ^a,b^	proteases, lipases, flavor compounds, pigments, growth factors, bacteriocins	[11,16,17,23,47,60,61,65,78,89,91,92,99,100,144,161,172,173]
Actinomycetales	*Dermabacteraceae*	***Brachybacterium***	facultative anaerobic	3.6–7.3% ^a^	flavor compounds, pigments	[11,16,17,32,60,92,157,172,173]
Actinomycetales	*Corynebacteriaceae*	***Corynebacterium***	facultative anaerobic	1.2–70% ^a,b^	esterases, lipases, flavor compounds	[16,23,60,65,89,91,100,143,144,172,173]
Actinomycetales	*Microbacteriaceae*	***Microbacterium***	facultative anaerobic	0.9% ^a^	flavor compounds, lactic acid, pigments, growth factors	[16,60,172,173]
Actinomycetales	*Microbacteriaceae*	***Leucobacter***	aerobic	0.08% ^a^	not known	[60,173]
**Proteobacteria**						
Oceanospirillales	*Halomonadaceae*	***Halomonas***	facultative anaerobic	2.5–29.1% ^a,b^	>not known	[46,47,60,92,143,144,161,172,173]
Enterobacterales	*Enterobacteriaceae*	***Enterobacter***	facultative anaerobic	0.02% ^a^	proteases, flavor compounds, pigments	[60,172,173]
Enterobacterales	*Enterobacteriaceae*	***Proteus***	facultative anaerobic	6.3% ^b^	proteinases, lipases, flavor compounds	[146,158,172,173]
Pseudomonadales	*Moraxellaceae*	***Psychrobacter***	aerobic	3–9.9% ^a,b^	>flavor compounds	[46,60,65,89,92,100,143,144,158,159,161,171,173]
Alteromonadales	*Pseudoalteromonadaceae*	***Pseudoalteromonas***	aerobic	3.5–3.8% ^a^	extracellular enzymes, pigments	[60,65,92,100]

Determined by ^a^ high-throughput sequencing analysis, ^b^ culture-based analysis.

## 5. Cheese Smear Defects

Whereas the safety aspects of smear-ripened cheeses were extensively discussed in detail by previous reviews [7,9,11,49,50,53], this review is also focusing on the impact of the surface-smear microbiota on quality aspects of smear-ripened cheeses, which are of utmost importance for the cheese industry. Cheese smear defects can lead to product recall costs, put a stop to whole production processes and thus have a great impact on economics for the cheese industry [74].

The fermentation of milk to cheese leads to a durable food product, where the high nutritional value of milk originating from its high proteins, carbohydrates, fats, vitamins, essential amino acids and minerals is preserved [4,6]. The reduction of the moisture content by syneresis during cheese production, as well as the salting of cheese, is further reducing the water activity (a_w_), and represents an important hurdle for microbial growth [103]. However, specially adapted microorganisms can cope with hurdles such as low water activity values, organic acids or high salt content and also manufacturing errors or improper cheese surface smear development can induce defects for smear cheeses. The smear may develop patchy or non-uniform on the cheese surface [175]. Furthermore, softer smear cheese varieties feature in general a reduced shelf-life due to a higher water activity and a more favorable pH for microbial growth [176]. In the same way as an elevated ripening temperature contributes to an enhanced ripening, the storage temperature has a potential stimulatory effect on pathogens and microbial spoilers by directly affecting their growth rate, as well as accelerating enzyme-catalyzed reactions, thus demonstrating the need for cold storage [174].

Gas-producing bacteria may cause defects in the texture of smear cheeses which can also affect the cheese surface [74,177]. Early blowing in smear cheeses can be induced at the beginning of the fermentation process by the growth of coliform bacteria, which is favored by a low activity of starter lactic acid bacteria that causes a diminished acid production [177]. Late blowing during the long ripening of hard and semi-hard cheeses is usually induced by the metabolic activity and growth of butyric acid bacteria such as *Clostridium butyricum* and *Clostridium tyrobutyricum* which not only produce butyrate, but also CO_2_ and H_2_ [177,178]. Clostridia produce heat-stable endospores, which survive the pasteurization process. They may originate from soil, silage, or the cow environment, where they enter the milk [178,179]. Thus, beside technical procedures such as microfiltration and bactofugation a good farming practice is important to achieve a low spore contamination of raw milk [179]. Further texture defects can also include surface crystallization of brushite, calcite, ikaite, and struvite and demineralization of calcium, magnesium, and phosphorus in smear cheeses [180]. Usually not harmful, such crystal structures can remind consumers of mold spoilage and may result in complaints and avoidance of this type of cheese [74]. Furthermore, light-induced oxidation processes of proteins and lipids may result in discoloration and off-flavor development [181,182,183,184].

Uncontrolled and extensive fungal and/or bacterial growth on the cheese surface during the cheese ripening process may cause smear defects that result in considerable economic losses to the dairy industry or may even provide a potential health risk for the consumer [177]. Slow growth or intensive development of the yeast *Geotrichum candidum* can induce a so-called ’toad skin’ defect [177]. Another type of unwanted rind defect is red-brown pigmentation, which is occasionally found in smeared-ripened cheeses such as Fontina, Tilsit, and other cheese types, such as Emmental, Provolone, Grana, Romano, and Parmesan. This defect is also called ‘bankrot’ or ’shelf reddening’ and is thought to be caused by an excessive development of typical members of the surface microbiota present on the rind, whereas ink or red discoloration in cheese can be induced by *Thermus* species (*Thermus thermophilus* formerly known as *Flavobacterium*) but also by thermophilic lactobacilli and propionic acid bacteria [74,185]. Sometimes, the red coloration permeates through to the initial part of the cheese under the rind, making the cheese unappealing and unacceptable for consumers, even if odor and taste are not affected by this defect [186]. The characterization of the microbiota, related to the red-brown defect in cheese and on wooden shelves used during the ripening of cheese by Guzon and colleagues showed that the red-brown defect in smear-ripened cheese was associated with a change in the microbial population that is usually present in the cheese and on the wooden shelves used during ripening [186]. In particular, Actinobacteria and *Debaryomyces* were detected in high concentration on red-browning shelves and, therefore, considered to be associated with the emergence of the red-brown defect in this type of cheese [186]. Furthermore, the efficacy of different cleaning measures and treatments to decrease the microbial counts on wooden shelves was verified in order to prevent the development of the red-brown defect during cheese ripening. Kamelamela and colleagues investigated the cause for the development of a distinctly purple rind during the ripening of a washed-rind cheese produced from raw milk in the United States [187]. The isolation of Gram-negative *Proteus* and *Psychrobacter* spp. led to the assumption that members of these genera were responsible for the production of the purple-red pigments. This assumption could be confirmed by experimentally applying microbial communities with or without *Proteus* and *Psychrobacter,* which demonstrated that these Proteobacteria cause the purple coloration of the cheese rinds. Their results suggested that the purple rind defect is not limited to certain cheese types. Other cheeses may also exhibit purple pigmentation when high numbers of *Proteus* and possibly *Psychrobacter* are present and certain conditions are provided. Consequently, the observation of pigment production by *Proteus* and *Psychrobacter* strains isolated from other cheese rinds suggested that the purple rind defect has the potential to be widespread in surface-ripened cheeses [187]. However, in another type of cheese from Spain called Cabrales, a traditional, blue-veined Spanish cheese made from raw milk, a prodigiosin-producing strain of *Serratia marcescens* was identified to be responsible for the development of this defect. Although the source of *S. marcescens* was not undoubtedly identified, the requirement of high numbers of bacterial cells for the red defect to develop in experimental cheeses and the occasional presence of prodigiosin-producing *S*. *marcescens* strains in the milk destined for Cabrales manufacture strongly suggested that the cheese milk is the source for this bacterium [188]. Furthermore, pink and red discoloration defects, as well as brown pigments, are also associated with the growth of certain yeast species, such as *Rhodotorula* spp. or *Yarrowia lipolytica* [104,189]. It is assumed that these browning reactions are due to high free tyrosine levels produced by tyrosinase-containing yeast such as *Yarrowia* species [177]. Besides discoloration defects, yeast can also induce off-flavors, softening, gas production, and swollen cheese packages [190]. Further smear surface defects causing spots in differing colors such as yellow and green-fluorescent spots, but sometimes also brown spots, may be caused by the activity of various *Pseudomonas* species, such as *Pseudomonas fluorescence*. This type of defect is often associated with off-flavors such as bitterness, rancid, putrid, or pungent, running paste, and generally an improper development of the typical ripening flora [74,147,177]. Further contaminating microorganisms leading to discoloration of cheese might originate from primary starter bacteria (e.g., *Lactobacillus* spp.), *Candida* spp., or enterococci [191]. A common taste defect is caused by an excess of sulfurous flavors or fruity flavors. Fruity flavors usually are off-flavors due to the production of ethyl esters by *Lactococcus lactis*. Sulphur compounds produced by common smear bacteria, such as coryneform bacteria, are key flavors in many smear-ripened cheeses. However, they show a low flavor threshold and may rapidly affect the flavor adversely [74]. These examples demonstrate that color and flavor defects are widespread in various types of cheese and that different microorganisms may be responsible for the occurrence of these defects in different cheese varieties. Thus, the difference between a beneficial or spoilage outcome seems to be determined on a strain-level [190]. It needs to be mentioned that fermentation failures at the beginning of cheese production will usually lead to a subsequent improper ripening process that may induce smear cheese defects. Besides various other reasons, such fermentation failures can be caused by bacteriophage infections [192].

Growth of mold on smear cheese represents a smear defect, which not only negatively impacts the flavor, taste, and color of the cheese but might also lead to the formation of toxic secondary metabolites such as mycotoxins [175,177,193]. Therefore, storage of cheese under refrigerated conditions is thought to diminish the growth of mold. However, psychrotolerant mold, which is able to grow at low oxygen tension, can still grow on the cheese surface [193]. In general, the major parameters that affect mold growth on the surface of cheese comprise temperature conditions, carbon dioxide concentrations and the salt content [194]. In particular smear-ripened cheeses matured for longer times bear a higher risk for spoilage by mold [175]. To minimize the growth of mold for the preservation of the cheese quality and to increase the shelf life of the cheese, oxygen needs to be reduced to inhibit extensive fungal and bacterial spoilage or oxidation of the cheese [195,196,197]. The predominant mold identified as spoiling microorganisms on the surface of smear-cheese are *Penicillium* species like *P*. *commune*, *P*. *glabrum*, and *P*. *nalgiovense,* whereas *P. verrucosum*, *P. solitum*, *P. roqueforti*, *P. discolor*, *P. crustosum,* and *P. palitans* together with *Aspergillus versicolor* were only frequently reported [175,193,194]. *Epicoccum* spp., which are widely recognized as plant pathogens, have been recently identified as the causative agent for reddish-brown spot defects on cheese [188]. *Epicoccum* sp. were found to colonize the whole cheesemaking environment, thus revealing a difficult task for the elimination as a spoilage source in order to render a hygienic production environment [188]. Besides poor factory hygiene, mold defects can also develop due to poor sanitation of ripening areas, airborne mold spores, movement of personnel, aerosol generation, mold-contaminated brine, poor smear development, ‘old-young smearing’ application, and inadequate humidity or surface treatment [175]. According to several studies, the level of mycotoxin contamination is, in general, low, even when visible mold growth occurs on cheese [193,194]. However, safe consumption of such cheese cannot be guaranteed, as various filamentous fungi can produce mycotoxins, and for aflatoxins, it was demonstrated that they may penetrate into the cheese wheel up to a depth of 4 cm from the surface [193]. Therefore, strict compliance with hygienic standards, application of pure starter cultures, control of cheese smear contamination, and packaging under modified atmospheric conditions might be applied to reduce unwanted growth of mold on cheese. However, the growth of mold such as *Fusarium domesticum* can have a favorable effect by reducing sticky surface smear defect [164].

Packaging of matured smear cheese is usually performed to prevent microbial and chemical quality deteriorations and to better facilitate the handling and marketing of the products [198]. Due to the requirements of retailer logistics and the increasing vending of cheese portions in self-service shelves of supermarkets, fully ripened red-smear cheeses are increasingly distributed and stored as vacuum film-prepackaged cheese wheels or portions. However, prepackaging of cheese in plastic foil is often accompanied by unwanted changes in the olfactory characteristics and a marked deterioration in the cheese surface smear quality. Such cheeses develop a wet, sticky, and smudgy surface smear and exhibit an unpleasant off-flavor upon opening of the packed cheese. Previous studies ruled out a contribution of yeast and pointed to alterations in the composition or the metabolic activity of the smear microbiota due to changes in the physicochemical characteristics of the cheese smear after vacuum film-prepackaging as a reasonable explanation [172,194,199]. Studies investigating this issue revealed several bacteria with the potential to contribute to smear defects by off-flavor or exopolysaccharide production, but they did not provide evidence for the contribution of a distinct microbial group. In contrast, the obtained results suggested a contribution of various microorganisms and their metabolic activity due to the altered conditions provided by vacuum film-prepackaging [172]. In a later study on the same smear cheese defect, a molecular approach using next-generation sequencing of amplified 16S rRNA gene fragments was applied to identify potential alterations in the composition of the cheese smear microbiota [173]. An unexpectedly high diversity of 132 different genera from the domains Bacteria and Archaea was detected on the analyzed cheese surfaces. These comprised typical smear microorganisms as well as microorganisms so far not associated with cheese, but related to the milk, farm, and cheese dairy environment. A 16S ribosomal RNA based analysis from total RNA identified the major metabolically active populations of the cheese surface smear microbiota as Actinobacteria of the genera *Corynebacterium*, *Brevibacterium*, *Brachybacterium*, and *Agrococcus*. Comparison of data on a higher phylogenetic level revealed increased proportions of Proteobacteria and Bacteroidetes in the smear of foil-prepacked cheese and particularly in samples showing defective smear, whereas staphylococci showed an opposite trend and turned out to be strongly decreased in defective smear. Again, the observed shifts in the microbial composition of samples from defect surface smear did not identify a single microbial species but rather suggested a combined contribution of a decrease in typical smear organisms like staphylococci, together with an increase in members of Proteobacteria that might substantially contribute to the observed negative organoleptic properties of the surface smear after the prepacking of the cheese in plastic foil [173].

Overall, cheese quality defects may be influenced by seasonality and the variability of the milk composition applied for cheese production, variations in cheese production process parameters, as well as the nature and number of microorganisms applied as starter cultures or the natural development of non-starter microorganisms on the cheese surface [74,175]. In addition to traditional culture-based approaches, state-of-the-art nucleic acid-based high-throughput analysis techniques allow today the investigation of microbe-related cheese quality defects caused by alterations of the complex multi-species matrix of the cheese surface microbiota more in-depth than ever before [74].

## 6. Conclusions and Prospects

The smear microbiota present on the rind of surface-ripened cheese constitutes a complex microbial consortium encompassing prokaryotic, eukaryotic, and viral populations. Without the contributions of this consortium during production and ripening, surface-ripened cheeses would not develop as consumers are accustomed to, nor would they meet their expectations associated with smear-ripened cheeses. Therefore, the smear microbiota plays a pivotal role in shaping the distinctive flavor and appearance of this cheese variety while also directly influencing its safety and shelf life. Consequently, this review delves into the microbial diversity of the surface smear by discussing the relevant microbial groups and their important contribution to the cheese ripening process, which directly impacts the quality and safety of smear-ripened cheeses. Although classical, culture-based approaches had previously provided substantial knowledge regarding the composition of the microbial communities on smear-ripened cheeses, the widespread adoption of molecular high-throughput sequencing techniques in the past decade has significantly verified and enhanced our understanding of the surface smear microbiota’s detailed composition at an unprecedented extent. The present and future application of further developed genomics, metagenomics, transcriptomics, and metatranscriptomics analysis tools will not only provide an answer to the question “Who is there?” but also shed light on “What are they doing there?”. Thereby providing a valuable contribution to a detailed description of the roles of the different surface smear community members.

Despite the current progress, the specific activities and origin of many members of the smear microbiota remain unknown. Profound knowledge about their inter- and intra-species interactions within the surface smear ecosystem is still lacking, even though this knowledge would be of utmost value for the cheese industry and researchers to improve the quality and safety of this cheese variety by counteracting pathogens and preventing cheese quality defects. As an example, the investigation of cheese smear defects, such as the formation of excessively humid and slimy smear with an off-odor flavor after vacuum film-prepackaging, revealed a complex etiology that is not entirely understood yet. The formation of defective smear could not be attributed to a particular community member or a distinct subpopulation of the smear microbiota, thus emphasizing the importance of a comprehensive understanding of the complex surface smear ecosystem and its environmental conditions for preventing microbe-related cheese defects. The extensive adoption of molecular high-throughput sequencing techniques has the potential to unveil the putative mechanisms of interaction among the diverse microbial populations within the smear microbiota. This, in turn, will deepen our understanding of the composition, diversity, and structure of the surface smear microbiota, along with shedding light on the potential interactions among key community members. The increased understanding of the intricate surface smear ecosystem may serve as an important prerequisite for refining the cheesemaking and ripening process with direct implications for the quality and safety of smear-ripened cheese.

## Figures and Tables

**Table 1:** Overview of smear-ripened cheese varieties produced primarily in European countries ^a^. (based on McSweeney et al., 2004 [10] and adapted from Lücke and Zangerl, 2004 [38]).

Soft and Semi-Soft Cheese	Semi-Hard Cheese	Hard Cheese
Brick (US)	Appenzeller (CH)	Bergkäse (AT, CH, DE)
Epoisses (FR)	Danbo (DK)	Comté (FR)
Langres (FR)	Havarti (DK)	Esrom (DK)
Limburger (DE)	Jurakäse (CH)	Gruyère (CH, FR)
Livarot (FR)	Kenhem (NL)	
Maroilles (FR)	Mutschli (CH)	**Ripened acid curd cheese**
Mont d’Or (FR)	Raclette (CH, FR)	Harzer, Mainzer (DE)
Münster (DE, FR)	Ridder (NO)	Quargel (CZ, DE, AT)
Port de Salut (FR)	Salers (FR)	
Romadur (DE, BE)	St. Paulin (Suisse) (CH, FR)	**Smear-ripened cheese with mold**
Saint Nectaire (FR)	Tête de Moine (CH)	Chaumes (FR)
Trappiste (FR)	Tilsit (CH, DE)	Pont l’Évêque (FR)
Vacherin Mont-d’Or (CH)	Vacherin Fribourgeois (CH)	Reblochon (de Savoie) (FR)
	Winzerkäse (CH)	Taleggio (IT)

^a^ Country of origin in parenthesis: AT, Austria; CH, Switzerland; CZ, Czech Republic; BE, Belgium; DE, Germany; DK, Denmark; FR, France; IT, Italy; NL, Netherlands; NO, Norway; US, United States of America.

## Data Availability

Data is contained within the article.
